# Viral coinfection in acute respiratory infection in Mexican children treated by the emergency service: A cross-sectional study

**DOI:** 10.1186/s13052-015-0133-7

**Published:** 2015-04-18

**Authors:** Jahaziel Diaz, Jaime Morales-Romero, Gustavo Pérez-Gil, Martín Bedolla-Barajas, Netzahualpilli Delgado-Figueroa, Rebeca García-Román, Omar López-López, Evelyn Bañuelos, Cristal Rizada-Antel, Roberto Zenteno-Cuevas, Ángel Ramos-Ligonio, Clara Luz Sampieri, Luis Gustavo Orozco-Alatorre, Silvia I Mora, Hilda Montero

**Affiliations:** Instituto de Salud Pública, Universidad Veracruzana, Av. Luis Castelazo Ayala s/n., Col. Industrial Ánimas, 91190 Xalapa, Veracruz México; Centro de Ciencias Biomédicas, Universidad Veracruzana, Av. Luis Castelazo Ayala s/n., Col. Industrial Ánimas, 91190 Xalapa, Veracruz México; Hospital Civil de Guadalajara “Dr. Juan I. Menchaca”, Salvador Quevedo y Zubieta 750, Col. La Perla, 44100 Guadalajara, Jalisco México; Facultad de Química Farmacéutica Biológica, Universidad Veracruzana, Lomas del Estadio s/n, Col. Zona Universitaria, 91000 Xalapa, Veracruz México; Facultad de Ciencias Químicas, Universidad Veracruzana, Prolongación de Oriente 6, 1009, Col. Rafael Alvarado, 94340 Orizaba, Veracruz México; Unidad de Procedimientos Preparativos y de acceso a servicios de Proteómica, Instituto de Investigaciones Biomédicas, Universidad Nacional Autónoma de México, Av. Universidad, Col. Ciudad Universitaria, 04510 Distrito Federal, México

**Keywords:** Respiratory viruses, Coinfection, Acute respiratory infections, Children, Overcrowding, Emergency service, Metapneumovirus

## Abstract

**Background:**

Acute respiratory infections (ARIs) cause illness. Children under five years of age are highly vulnerable to these infections. Viral coinfection or multiple viral infection is a variable that can have a significant impact on the evolution of these diseases.

**Methods:**

This cross-sectional study was carried out in Mexican children (under five years of age) who had an ARI and who were treated by an emergency service in a hospital in Guadalajara, Jalisco, Mexico. The viral etiology, as well as the presence of multiple viral infections, was determined. A structured questionnaire was used to obtain demographic and clinical information. Odds ratio (OR) was calculated, and univariate and multivariate analyses using logistic regression were performed.

**Results:**

In the study population, metapneumovirus (hMPV) was the most frequent virus (22%), followed by adenovirus (hAD) (16%), respiratory syncytial virus (RSV) (14%), rhinovirus (hRV) (12%), bocavirus (hBoV) (9%), influenza virus (IF) (7%), and parainfluenza (PIF) (4%). The frequency of viral coinfections was 31.62%, and multiple logistic regression analysis revealed that hMPV, RSV, PIF, and hBoV were independently associated with multiple viral infection. No difference was found in the clinical manifestation of children with simple and multiple infections. Simple hMPV infection was associated with patients who presented with severe ARI. Using a multivariate analysis, we found that overcrowding is associated with coinfection when the viral etiology was hRV (OR = 2.56, 95% confidence interval (CI) 1.07 to 6.13), IF (OR = 2.56, 95% CI 1.07 to 6.13), PIF (OR = 2.96, 95% CI 1.15 to 7.65), hAD (OR = 2.56, 95% CI 1.07 to 6.13), and hBoV (OR = 2.9, 95% CI 1.14 to 7.34).

**Conclusions:**

Viral coinfections are frequent in children requiring treatment by an emergency service. However, the severity of ARI is similar to that of children with a simple infection. The hMPV is common and may confer a significant disease burden in the Mexican population. Finally, overcrowding is a housing characteristic that favors the development of coinfections.

**Electronic supplementary material:**

The online version of this article (doi:10.1186/s13052-015-0133-7) contains supplementary material, which is available to authorized users.

## Background

Acute respiratory infections (ARIs) are a public health problem due to their high morbidity. According to the World Health Organization (WHO), of the total number of new cases of pneumonia in the world, about 7% to 13% require hospitalization [1], and pneumonia results in approximately 1.4 million deaths per year in children under five years of age, mainly in developing countries [2].

Several microorganisms can cause ARI. However, most of them are of viral etiology [3,4]; respiratory syncytial virus (RSV), rhinovirus (hRV), influenza virus (IF), adenovirus (hAD), and parainfluenza (PIF) are the most frequently involved viruses (reviewed in [4,5]). All of these viruses are of clinical importance, and RSV has been found to be one of the most common viruses in ARI. Additionally, RSV infection has been associated with a more aggressive behavior and an increased need for hospitalization [6-9]. Given the way these respiratory viruses are spread and the diversity of types and variants [3,10], it is not surprising that sometimes the same patient is infected with more than one virus. Therefore, many research groups have tried to determine the consequence of multiple infections as clinical manifestations worsen. However, the results show discrepancies, because in some studies severe ARI occurs mainly in individuals with simple infections (reviewed in [11]).

The etiologic factor is not the only one influencing ARI complications; environmental and socioeconomic factors also impact on disease progression. A meta-analysis found that low birth weight, lack of exclusive breastfeeding, and overcrowding, among other factors, are associated with severe ARI (reviewed in [12]).

The purpose of this study was to determine the prevalence of respiratory viruses in Mexican children who were under five years of age, had an ARI, and were admitted to the emergency department of a single institution in Guadalajara, Jalisco, Mexico. It also evaluates the relationship between viral coinfection with clinical manifestations and some possible risk factors related to demographic and housing characteristics.

## Methods

### Study design

This cross-sectional study was carried out in Mexican children with ARI from December 2012 to April 2013 in a secondary care hospital located in Guadalajara, Jalisco, Mexico. The study was approved by the research committee of the hospital. The parents of the recruited infants were informed about the characteristics of the study and provided written informed consent for a sample to be taken and analyzed. A structured questionnaire was used to obtain demographic and clinical information.

### Subjects

In total, 162 samples from children under five years of age were obtained from a pediatric emergency service from December 2012 to April 2013 (corresponding to winter and spring). The inclusion criteria were age of less than five years, upper or lower respiratory tract manifestations of ARI, or both. The patients were divided into two groups according to severity of ARI: mild-moderate and severe. The criterion for determining severe ARI was that the patient had at least one sign of respiratory distress according to Official Mexican Norm NOM-031-SSA2-1999, which pertains to the care of children’s health: dyspnea, apnea, wheezing, or intercostal retraction [13]. Mild-moderate ARIs were all ARIs without respiratory distress. In this study, the patients had no fatal outcome.

### Definitions

ARI was defined according to the Mexican Official Norm as infectious disease caused by microorganisms that affect the respiratory system during a period of shorter than 15 days. The ARI should have been diagnosed by the attending physician [13].

Overcrowded housing was indicated when the quotient obtained by dividing the number of household members by the number of rooms available to sleep in was greater than or equal to 3 [14].

History of breastfeeding was assessed as duration in months and was divided into two groups: greater than or equal to 6 months versus less than 6 months. There was no further questioning about whether breastfeeding was exclusive.

Comorbidity was defined as the presence of one or more diseases or conditions other than those of primary interest [15].

Nutritional status was evaluated by body mass index as underweight, normal weight, and excess weight (overweight or obesity) according to Child Growth Standards, (World Health Organization) [16].

### Clinical samples

The nasal swabs were collected with sterile rayon-tipped applicators (Puritan Medical Products Co. LLC, Guilford, ME, USA) in accordance with WHO recommendations [17]. The swab was placed into a tube that contained Leibovitz viral transport medium with 100 IU/mL penicillin (American Type Culture Collection, Manassas, VA, USA), 0.25 μg/mL amphotericin B, and 0.01 mg/mL streptomycin (American Type Culture Collection). The samples were maintained at 4°C and transported at this temperature from the hospital in Guadalajara, Jalisco, to the virology laboratory of the Instituto de Salud Pública, Universidad Veracruzana (Public Health Institute, University of Veracruz), where the samples were processed and analyzed.

### Virus detection

Viral genomes were extracted with a PureLink viral RNA/DNA kit (Invitrogen Corporation, Carlsbad, CA, USA) in accordance with the instructions of the manufacturer. A screening for detecting bocavirus (hBoV) [18], hAD [19], RSV [20], hRV [21], metapneumovirus (hMPV) [22,23], influenza type A (IF), and PIF was carried out by reverse transcription-polymerase chain reaction (RT-PCR) or PCR by means of an AccessQuick™ RT-PCR System (Promega Corporation, Madison, WI, USA) in accordance with the instructions of the manufacturer and with previously described primers and methods; the modifications to these methods are indicated in Additional file 1. The PIF primers were designed by our group, and the IF primers were designed and provided by Susana López and her group (National Autonomous University of Mexico).

### Statistical analysis

Associations between different variables were assessed by the chi-square test or Fisher test where necessary. Statistical significance was defined as a *P* value of less than 0.05. Odds ratios (ORs) and 95% confidence intervals (CIs) were calculated by logistic regression. Statistical analysis was performed by using SPSS software (PASW Statistics for Windows, Version 18.0; SPSS Inc., Chicago, IL, USA) and StatCalc (Epi Info 7; Centers for Disease Control and Prevention, Atlanta, GA, USA).

## Results

### Characteristics of the participants

One hundred sixty-two patients were included in this study. The characteristics of the participants were analyzed. The average age of the population was 18.4 months (1.87 to 34.85 months), 28.4% were not more than 6 months old, and 58.6% of participants were male.

Clinical signs of an ARI were evaluated according to the data obtained from the questionnaire. All participants had nasal discharge and cough, and 67.3% had fever.

In regard to the housing characteristics, 8% of the participants lived in houses where they cooked with firewood, 38.9% reported having lived in overcrowded conditions, and less than 2% lived in a house with a soil floor. Furthermore, 88.8% had been breastfed at least once.

### Viral etiology

Of the samples tested, 57% were positive for a virus. The viruses most frequently detected were hMPV (22%), hAD (16%), RSV (14%), and hRV (12%). hBoV (9%), IF (7%), and PIF (4%) were detected at a lower frequency (Table 1).

**Table 1:** **Viral detection by polymerase chain reaction**

**Viruses**	**Frequency n = 162**	**Prevalence (95% CI)**
Viral etiology	93	0.57 (0.5-0.6)
hMPV	36	0.22 (0.1-0.3)
hAD	26	0.16 (0.1-0.2)
RSV	23	0.14 (0.1-0.2)
hRV	19	0.12 (0.1-0.2)
hBoV	16	0.09 (0.04-0.1)
IF	11	0.07 (0.03-0.1)
PIF	7	0.04 (0.01-0.1)

CI: confidence interval; hMPV: human metapneumovirus; hAD: human adenovirus; RSV: respiratory syncytial virus; hRV: human rhinovirus; hBoV: human bocavirus; IF: influenza virus; PIF: parainfluenza virus.

### Viral etiology and severity of the acute respiratory infection

The presence of each virus in mild-moderate or severe ARI was analyzed. hMPV was found to be associated with the severe ARI group (OR = 4.0, 95% CI 1.33 to 12.06). Other viruses we analyzed showed no association with any of the groups.

### Viral coinfections

The presence of more than one virus was evaluated in the sample coming from the same subject. Simple infections were the most frequent, whereas multiple infections occurred in 31.62% of patients with ARI of viral etiology. hMPV was the most frequent virus in coinfections (58.8%), followed by hAD and RSV (38.2% each).

A univariate analysis showed that only four of the seven viruses studied were significantly associated with multiple infection; PIF was the highest coinfection risk (OR = 12.42, 95% CI 1.4 to 108.3), followed by hBoV (OR = 5.16, 95 % CI 1.6 to 16.6), hMPV (OR = 3.8, 95% CI 1.6 to 9.4), and RSV (OR = 3.03, 95% CI 1.15 to 8.0). hAD, IF, and hRV showed no association (Table 2).

**Table 2:** **Comparison of viral etiology between subjects with multiple and simple infection**

	**Coinfection n = 34**	**Simple infection n = 59**	**OR (95% CI)**	***P*** **value**
**Viral etiology, n (%)**				
hMPV	20 (58.8)	16 (27.1)	3.8 (1.6-9.4)	0.003
hAD	13 (38.2)	13 (22.0)	2.19 (0.9-5.5)	0.094
RSV	13 (38.2)	10 (16.9)	3.03 (1.15-8.0)	0.022
hRV	10 (29.4)	9 (15.3)	2.31 (0.8-6.4)	0.103
hBoV	11 (32.4)	5 (8.5)	5.16 (1.6-16.6)	0.003
IF	6 (17.6)	5 (8.5)	2.31 (0.7-8.3)	0.187
PIF	6 (17.6)	1 (1.7)	12.42 (1.4-108.3)	0.009

Proportions were compared by using the chi-square test. OR: odds ratio; CI: confidence interval; hMPV: human metapneumovirus; hAD: human adenovirus; RSV: respiratory syncytial virus; hRV: human rhinovirus; hBoV: human bocavirus; IF: influenza virus; PIF: parainfluenza virus.

In the study population, 24.18% of cases of dual infection and 7.44% of triple infection were found. In regard to dual infections, the highest combination was observed between RSV-IF (4.65%). In the triple coinfections, the most common combination was hMPV-hAD-hBoV (2.79%) (Table 3).

**Table 3:** **Description of the coinfection type**

**Coinfection**	**Frequency n = 93**	**Percentage**
Dual infection	26	24.18
RSV-IF	5	4.65
hMPV-hRV	4	3.72
hMPV -RSV	4	3.72
hMPV -hAD	4	3.72
hMPV –hBoV	2	1.86
hRV-RSV	2	1.86
hRV-hBoV	1	0.93
hMPV-PIF	1	0.93
PIF-RSV	1	0.93
PIF-hBoV	1	0.93
PIF-hAD	1	0.93
Triple infection	8	7.44
hMPV -hAD-hBoV	3	2.79
hMPV -hAD-PIF	2	1.86
hRV-hAD-hBoV	2	1.86
hRV-RSV-IF	1	0.93
Total coinfection	34	31.62

hMPV: human metapneumovirus; hAD: human adenovirus; RSV: respiratory syncytial virus; hRV: human rhinovirus; hBoV: human bocavirus; IF: influenza virus; PIF: parainfluenza virus.

### Association between clinical and demographic characteristics with coinfection

A univariate analysis was carried out to find the association between coinfection and the following variables: age, sex, using wood as fuel in the house, overcrowding, having a house with a soil floor, and breastfeeding. Age of less than or equal to 6 months and living in overcrowded conditions increased the risk for multiple infection (OR = 3.04, 95% CI 1.08 to 8.6 and OR = 2.6, 95% CI 1.1 to 6.1, respectively). The rest of the demographic variables showed no significant association.

In regard to clinical features, the presence of comorbidity, nutritional state, and severity of ARI were evaluated. We found no significant differences (Table 4).

**Table 4:** **Demographic and clinical characteristics of subjects with multiple or simple infection**

	**Coinfection n = 34**	**Simple infection n = 59**	**OR (95% CI)**	***P*** **value**
**Demographic, n (%)**				
Age of ≤ 6 months	11 (32.4)	8 (13.6)	3.04 (1.08-8.6)	0.03
Sex, male	20 (58.8)	33 (55.9)	1.1 (0.5-2.6)	0.79
Use of wood biofuel	6 (17.6)	4 (6.8)	2.9 (0.8-11.3)	0.10
Overcrowding	18 (52.9)	18 (30.5)	2.6 (1.1-6.1)	0.03
Floor other than soil	34 (100)	57 (96.6)	-	-
Without breastfeeding	5 (15.6)	7 (11.9)	1.37 (0.4-4.7)	0.61
Breastfeeding < 6 months*	1 (11.1)	6 (17.6)	0.58 (0.06-5.58)	0.99
The patient was born as a twin	0 (0)	1 (1.7)	-	0.44
**Clinical, n (%)**				
Chronic disease	2 (5.9)	8 (13.6)	0.39 (0.1-1.9)	0.25
Severe ARI**	28 (82.4)	45 (76.3)	1.45 (0.50-4.22)	0.49
Nutritional status*** Underweight Excess weight‡ Normal weight	6 (30.0) 3 (15.0) 11 (55.0)	8 (18.6) 15 (34.9) 20 (46.5)	1.36 (0.38-4.95) 0.36 (0.09-1.54) 1.0	0.64 0.16

Proportions were compared by using the chi-square test. CI: confidence interval; OR: odds ratio.

*Only in children who are more than 6 months old.

**Severe acute respiratory infection (ARI) was defined as respiratory distress (Official Mexican Norm NOM-031-SSA2-1999).

***Nutritional status assessed by body mass index according to Child Growth Standards (World Health Organization). There were 30 children in whom it was not possible to measure weight or height.

‡Overweight or obesity was included in excess weight.

### Multivariate analysis of factors associated with coinfection

A multivariate analysis was conducted in order to identify the risk factors for viral coinfection by making a stratification according to the etiological agent identified. Infection with hMPV, RSV, PIF, and hBoV showed significant association in the adjusted model. Overcrowding was a variable that remained associated in the adjusted analysis in those cases of infection with hRV (OR = 2.56, 95% CI 1.07 to 6.13), IF (OR = 2.56, 95% CI 1.07 to 6.13), PIF (OR = 2.96, 95% CI 1.15 to 7.65), hAD (OR = 2.56, 95% CI 1.07 to 6.13), and hBoV (OR = 2.9, 95% CI 1.14 to 7.34). The rest of the variables that had shown no significant association in the univariate analysis did not show one in the multivariate analysis either, except for age of less than 6 months, which was associated with infection with PIF and multiple infections (OR = 3.33, 95% CI 1.07 to 10.39) (Table 5).

**Table 5:** **Multivariate analysis of identified risk factors for coinfections**

	**Non-adjusted model**			Adjusted model*		
	**OR**	**95% CI**	***P***	**OR**	**95% CI**	***P***
**RSV**	2.8	1.02-7.68	0.045	3.03	1.15-8.00	0.025
Age < 6 months	2.50	0.80-7.90	0.12	-	-	-
Male	1.10	0.43-2.79	0.84	-	-	-
Overcrowding	2.53	1.01-6.34	0.047	-	-	-
**hMPV**	3.53	1.40-8.96	0.008	3.84	1.57-9.37	0.003
Age < 6 months	2.14	0.66-6.96	0.21	-	-	-
Male	0.97	0.37-2.52	0.95	-	-	-
Overcrowding	2.6	1.02-6.62	0.046	-	-	-
**hRV**	2.43	0.82-7.21	0.110	-	-	-
Age < 6 months	2.45	0.78-7.65	0.12	-	-	-
Male	1.26	0.50-3.20	0.63	-	-	-
Overcrowding	2.75	1.10-6.87	0.030	2.56	1.07-6.13	0.034
**IF**	2.05	0.54-7.78	0.29	-	-	-
Age < 6 months	2.58	0.84-7.97	0.10	-	-	-
Male	1.08	0.43-2.71	0.87	-	-	-
Overcrowding	2.57	1.04-6.34	0.041	2.56	1.07-6.13	0.034
**PIF**	17.72	1.9-165.22	0.012	17.80	1.92-165.30	0.011
Age < 6 months	3.35	1.06-10.60	0.040	3.33	1.07 - 10.39	0.038
Male	1.03	0.40-2.70	0.95	-	-	-
Overcrowding	2.95	1.13-7.67	0.027	2.96	1.15 - 7.65	0.025
**hAD**	2.38	0.89-6.31	0.083	-	-	-
Age < 6 months	2.83	0.90-8.84	0.074	-	-	-
Male	1.24	0.49-3.14	0.65	-	-	-
Overcrowding	2.64	1.06-6.57	0.037	2.56	1.07-6.13	0.034
**hBoV**	5.93	1.75-20.10	0.004	5.83	1.74-19.55	0.004
Age < 6 months	2.67	0.84-8.48	0.095	-	-	-
Male	1.06	0.41-2.74	0.91	-	-	-
Overcrowding	3.02	1.17-7.80	0.023	2.90	1.14-7.34	0.025

Odds ratio (OR) was obtained by logistic regression. *Adjusted model: variables used for adjusting do not show their OR. CI: confidence interval; hMPV: human metapneumovirus; hAD: human adenovirus; RSV: respiratory syncytial virus; hRV: human rhinovirus; hBoV: human bocavirus; IF: influenza virus; PIF: parainfluenza virus.

## Discussion

In Mexico, as in other parts of the world, ARIs have a high impact on morbidity, and children are highly vulnerable to these diseases [2,24].

In this study, we conducted an analysis of the viral etiology and clinical features of children admitted to the pediatric emergency service of a secondary care hospital, located in western Mexico.

In regard to viral detection, we found that hMPV was the most frequent virus in simple infections in the population we analyzed. This virus recently has been identified and has been associated with ARI in children and adults [25,26]. In Mexico, a few studies on the prevalence of hMPV have been conducted in the pediatric population, and the frequency has been reported to range from 6% to 20% [27-29]. In this study, this virus was detected in 22% of children, a value that is very similar to what has been reported in Mexico [28,29] and elsewhere (reviewed in [30]). Furthermore, our results contrast with those of other studies in which RSV ranks as the most frequent virus in the pediatric population with ARI [6,7]. The contrasts are probably due to the different temperatures (which are recorded by season) and the different geographical location of Mexico in comparison with those of other countries.

Interestingly, we found hAD to be the second most common virus. This virus is a cause of ARI and has been reported to have a frequency of about 3% in Mexican children from states other than Jalisco [31,32]. In this study, hAD was identified in 16% of patients, a value that is higher than what has commonly been reported in Mexican studies. In other countries, the prevalence of hAD varies from 1% to 20% [33-36]. Our results could be explained by the fact that the sample collection may have coincided with an outbreak of this virus. It is important to consider that genomic material of this virus has been found repeatedly in infants with recurrent episodes of ARI [37] and in children with asthma [38]. This is an important issue to investigate in future studies because infection with hAD may have health complications after the initial infection and this information is not known in the Mexican population.

In addition to determining the frequency of the viruses in each of the two groups in our study, we investigated whether a virus was associated with the severity of ARI. Our results showed that hMPV was associated with patients who presented with a severe ARI similar to that found in other populations [39-41]. Owing to the hMPV association with a greater severe clinical profile and its high frequency in our population, the results indicate that it is necessary to enhance surveillance for ARI in order to identify hMPV as an emerging virus, similarly to what is carried out with IF in the Mexican population.

The presence of more than one type of virus in the same patient has been an interesting topic that has led us to investigate the importance and clinical relevance of a coinfection. In some populations, coinfection with multiple respiratory viruses is a possible risk factor for complications of ARI. However, this has not occurred consistently in all of the studies [8,11,42]. The frequency of coinfection in our study was 31.62%, and according to our results, this did not create conditions for a different severity of ARI compared with those with a simple infection. Disagreements about the impact of a viral coinfection on the clinical manifestations may be due to the sample collection period, the amount and type of virus detected, viral detection technique, and the characteristics of the population.v On the other hand, we should consider that, owing to its cross-sectional design, our study did not follow the evolution of the clinical profile.

Little is known about viral coinfections and the variables influencing them. We analyzed some risk factors for infection with multiple viruses. Interestingly, by conducting a multivariate analysis, we found that certain types of viral etiology were significantly associated with the presence of coinfections: PIF, HBoV, hMPV, and RSV. Similar to what we found, RSV, hMPV, and hBoV have been reported as having a high frequency in infections with more than one virus [43-45].

Moreover, factors other than etiological agent may influence the development of ARI. In this regard, many research groups have investigated those factors that may be modifiable and that have an impact on the health of the population. Breastfeeding, exposure to smoke of wood or cigarettes, nutritional status, and variables related to housing have been reported as possible factors impacting the evolution of respiratory infections (reviewed in [12,46]). In this study, we evaluated breastfeeding, age of less than 6 months, chronic illness, floor type in the house, exposure to wood smoke, and overcrowding as possible factors related to viral coinfection. Of these, age of less than 6 months was found to be a risk factor for coinfection only when the disease is caused by PIF.

Furthermore, overcrowding, which in the univariate analysis had been identified as a factor independently associated with the presence of coinfection, kept this association in the multivariate analysis only when the ARI is caused by hRV, IF, PIF, hAD, and hBoV. Overcrowding is common in developing countries, where the presence of a large number of people in a single room used for sleeping favors the transmission of different pathogens, and children are the most vulnerable to acquire and develop the infection, and it was reported that this housing characteristic was a risk factor for ARI [47,48]. In a meta-analysis to find factors associated with severity of respiratory infections, it was found that overcrowding conferred a meta-estimated OR of 1.9 (95% CI 1.5 to 2.5) [12]. Interestingly, in a study conducted in refugee camps in Kenya, the frequency of respiratory viruses was determined, and hAD, RSV, hMPV, PIF, and IF (in descending order) were found to be the most prevalent in circumstances characterized by a great number of people in a single space [35]. It is noteworthy that most of the population in our study is located in one of the most populated metropolitan areas in Mexico, suggesting that overcrowding still occurs. Thus, the characteristics of housing continue to be a factor for health problems in the Mexican population, and overcrowding does not occur only in rural areas [14,49].

There are some limitations to our study. First, this research was conducted only from December 2012 to April 2013 (in Mexico, winter begins on December 20), and so we might be overestimating the frequency of some viruses if the study coincided with the peak incidence of any of them or we might be underestimating if it did not. Second, this study did not look for any bacteria in the samples. It has been found that some viruses bring on bacterial infections and this condition could influence these signs and symptoms present during infection [50]. This topic is very interesting and needs to be developed in future research. Third, the study included patients who went to the emergency department, which is usually required when patients develop complications of ARI. Therefore, to have a better understanding of the influence of viral etiology on the clinical spectrum of the disease, it would be interesting to study patients with different degrees of respiratory infection severity, including ambulatory patients from outpatient clinics and hospitalized patients as well as asymptomatic subjects, given that respiratory viruses have been found in children without symptoms of ARI [51]. In the future, it would also be desirable to conduct further studies that completely follow the clinical course of patients with ARI.

## Conclusions

In conclusion, viral coinfections have a high frequency in the population of Mexican children treated in the emergency department; in this population, overcrowding is a significant risk factor that allows contact of the host with multiple pathogens, but the coinfection depends on the type of virus occurring in the infection. hMPV is a common virus in the population we studied and is significantly associated with severe ARI, a situation that seems to create conditions for an increase in medical attention in the emergency department. Therefore, this virus could represent a major health burden among Mexican children.

## Additional file

Additional file 1:
**Methods PCR.**

## Abbreviations

ARI: Acute respiratory infection
OD: Odss ratio
hMPV: Human metapneumovirus
hAD: Human adenovirus
RSV: Respiratory syncytial virus
hRV: Human rhinovirus
hBoV: Human bocavirus
IF: Influenza virus
PIF: Parainfluenza virus
CI: Confidence interval
