# Supplementary material for: Viral coinfection in acute respiratory infection in Mexican children treated by the emergency service: A cross-sectional study
Source: Ital J Pediatr. 2015 Apr 18;41:33. doi: 10.1186/s13052-015-0133-7 (PMC4405868; doi:10.1186/s13052-015-0133-7)
Supplement: Additional file 1: — Methods PCR. [file 13052_2015_133_MOESM1_ESM.docx]

# Additional files

| **Virus** | **Gen** | **Primers** | | **Amplification condition** | | |
| --- | --- | --- | --- | --- | --- | --- |
|  |  | **Forward**  **5´-> 3’** | **Reverse**  **5´-> 3’** | **Cycles** | **Time**  **(00´00´)** | **Temperature**  **(^o^C)** |
| hAD  [16] | Hexon | CCTACGCACGATGTGACCACAGACCG | GTGTTGTAGGCAGTGCCGGAGTAGGG | - | 30´00’’ | 42 |
|  |  |  |  | - | 02´00’’ | 94 |
|  |  |  |  |  | 00´40´´ | 94 |
|  |  |  |  | 10 | 00´30´´ | 68 |
|  |  |  |  |  | 00´45’’ | 72 |
|  |  |  |  |  | 00´40’’ | 94 |
|  |  |  |  | 25 | 00´30´´ | 68 |
|  |  |  |  |  | 00´50´´ | 72 |
|  |  |  |  | - | 05´00 | 72 |
|  |  |  |  | - | ∞ | 4 |
| hBoV  [15] | NP1 | TAACTGCTCCAGCAAGTCCTCCA | GGAAGCTCTGTGTTGACTGAAT | - | 40´00’’ | 45 |
|  |  |  |  | - | 03´00’’ | 94 |
|  |  |  |  |  | 00´10´´ | 95 |
|  |  |  |  | 40 | 00´30´´ | 58 |
|  |  |  |  |  | 00´30’’ | 72 |
|  |  |  |  | - | 05´00’’ | 72 |
|  |  |  |  | - | ∞ | 4 |
|  |  | GCACTTCTGTTTCCCC | CGGACACCCAAAGTAG |  | 45´00’’ | 60 |
| hRV  [18] |  |  |  | - | 02´00’’ | 95 |
|  |  |  |  | 40 | 00´30´´ | 95 |
|  | UTR |  |  |  | 00´30´´ | 50 |
|  |  |  |  |  | 00´30’’ | 72 |
|  |  |  |  | - | 05´00’’ | 72 |
|  |  |  |  | - | ∞ | 4 |
| hMPV  [19] |  |  |  | - | 60´00’’ | 45 |
|  |  |  |  | - | 05´00’’ | 94 |
|  |  |  |  |  | 00´50´´ | 94 |
|  | F | CTTTGGACTTAATGACAGATG | GTCTTCCTGTGCTAACTTTG | 40 | 00´50´´ | 54 |
|  |  |  |  |  | 00´50’’ | 72 |
|  |  |  |  | - | 05´00’’ | 72 |
|  |  |  |  | - | ∞ | 4 |
|  |  |  |  | - | 02´00’’ | 94 |
| hMPV  [20] |  |  |  | - | 01´00´´ | 94 |
|  | F | CTGAACTAGCCAGAGCTGT | CATTGATTCCTGCTGCTGTGTC | 40 | 01´00´´ | 60 |
|  |  |  |  |  | 01´00’’ | 72 |
|  |  |  |  | - | 05´00’’ | 72 |
|  |  |  |  | - | ∞ | 4 |
|  |  | ggaacaagttgttgaggtttatgaatatgc | ttctgctgtcaagtctagtacactgtagt | - | 60´00 | 38 |
| RSV  [17] |  |  |  | - | 10´00’’ | 95 |
|  | N |  |  |  | 00´30´´ | 94 |
|  |  |  |  | 40 | 00´60´´ | 50 |
|  |  |  |  |  | 00´80’’ | 72 |
|  |  |  |  | - | 08´00’’ | 72 |
|  |  |  |  | - | ∞ | 4 |
|  |  |  |  | - | 30´00’’ | 50 |
|  |  |  |  | - | 15´00’’ | 95 |
|  |  |  |  |  | 00´30´´ | 94 |
| IF | M1 | CTTCTAACCGAGGTCGAAACG | CATGCAACTGGCAAGTGCACC | 40 | 00´30´´ | 50 |
|  |  |  |  |  | 00´60’’ | 72 |
|  |  |  |  | - | 07´00’’ | 72 |
|  |  |  |  | - | ∞ | 4 |
| PIF  (Nested PCR) | H-N | CACATCCTTGAGTGATTAAGT | CTGGAGATGTCCCGTAGGAG | - | 60´00’’ | 45 |
|  |  |  |  | - | 02´00’’ | 95 |
|  |  |  |  |  | 00´30´´ | 95 |
|  |  |  |  | 40 | 00´30´´ | 60 |
|  |  |  |  |  | 00´25’’ | 72 |
|  |  |  |  | - | 05´00’’ | 72 |
|  |  |  |  | - | ∞ | 4 |
|  |  | CACATCCTTGAGTGATTAAGT | CTGGAGATGTCCCGTAGGAG | - | 02´00’’ | 95 |
|  |  |  |  |  | 00´30’’ | 95 |
|  |  |  |  | 40 | 00´30´´ | 60 |
|  |  |  |  |  | 00´25´´ | 72 |
|  |  |  |  | - | 05´00’’ | 72 |
|  |  |  |  | - | ∞ | 4 |
| hMPV: human metapneumovirus; hAD: human adenovirus; RSV: respiratory syncytial virus; hRV: human rhinovirus; hBoV: human bocavirus; IF: influenza virus; PIF: parainfluenza virus. | | | | | | |
